# Is chromosome 9 loss a marker of disease recurrence in transitional cell carcinoma of the urinary bladder?

**DOI:** 10.1038/bjc.1998.365

**Published:** 1998-06

**Authors:** J. M. Bartlett, A. D. Watters, S. A. Ballantyne, J. J. Going, K. M. Grigor, T. G. Cooke

**Affiliations:** Glasgow University Department of Surgery, Glasgow Royal Infirmary, UK.

## Abstract

**Images:**


British Journal of Cancer (1998) 77(12), 2193–2198
© 1998 Cancer Research Campaign

### Is chromosome 9 loss a marker of disease recurrence in transitional cell carcinoma of the urinary bladder?

**JMS Bartlett[1], AD Watters[1], SA Ballantyne[1], JJ Going[2], KM Grigor[3] and TG Cooke[1]**

[1]Glasgow University Department of Surgery, Glasgow Royal Infirmary, Glasgow G31 2ER; [2]Glasgow University Department of Pathology, Glasgow Royal Infirmary, Glasgow G4 0SF, UK; [3]Edinburgh University Department of Pathology, Edinburgh EH8 9AG, UK

**Summary** Investigation of transitional cell carcinoma of the urinary bladder (TCC) patients classified by recurrence and/or progression has demonstrated that loss of chromosome 9, as detected by FISH analysis of the pericentromeric classical satellite marker at 9q12, occurs early. A total of 105 TCCs from 53 patients were analysed in situ by two independent observers for loss of chromosome 9 using quantitative fluorescence in situ hybridization (FISH). All 53 primary tumours were evaluated for chromosomes 9, 7 and 17. Normal ranges for chromosomal copy number were defined for normal skin epidermis and bladder epithelium. Values for chromosome 9 copy number outwith the range 1.51–2.10 (mean ± 3 × s.d. of normal values) were significantly abnormal. Twenty-five TCCs were detected with consistent monosomic scores. Of 89 TCCs, in which multiple tumour areas were analysed, 85 tumours (96%) demonstrated the same chromosome 9 copy number in all areas (2–6) analysed; only three tumours demonstrated heterogeneity for this locus. A total of 36% (12 out of 33) of patients with subsequent disease recurrence demonstrated loss of chromosome 9 in their primary and all subsequent TCCs analysed. Only a single patient ($n = 20$) with non-recurrent TCC showed loss of chromosome 9 ($P = 0.0085$). Of 53 primary tumours, eight showed significant elevation of chromosome 17. Of these patients, six demonstrated elevation in chromosome 7 copy number. No abnormalities were observed in non-recurrent patients. This study describes rapid quantitation of chromosomal copy number by FISH using a pericentromeric probe for chromosome 9 in TCC of the urinary bladder. Routinely fixed and processed material was evaluated without disaggregation. Strict quality control of FISH demonstrated that this technique was reproducible in a clinical environment and could be used to detect genetic changes relevant to patient outcome. It is proposed that loss of chromosome 9 from primary TCC of the urinary bladder identified patients at high risk of recurrence and possible progression.

**Keywords:** Fluorescence in situ hybridization; bladder; transitional cell carcinoma; chromosome 9; diagnostic; pathology; quantitation; monosomy; recurrence; survival

Transitional cell carcinoma of the urinary bladder (TCC) is the fourth commonest cancer of men in the UK, with a sex ratio of 3:1 (M/F) and increasing mortality and incidence (Anon, 1984; 1987). Patients with superficial TCC have a better prognosis (5-year survival 95%) than those with detrusor muscle invasion (35% 5-year survival; Anon, 1984; 1987). Low-stage and grade tumours represent 70% of primary TCCs, but frequent recurrence and risk of disease progression at recurrence mandate careful monitoring of such patients (McCredie, 1994; Ozen, 1994). Current management is by cystoscopy 3, 6 and 12 months after each recurrence, and annually thereafter (McCredie, 1994; Ozen, 1994). No predictive marker of recurrence or progression has yet been identified using conventional pathological staging and grading (UICC, 1978) or by molecular and biochemical techniques.

Investigations of TCC tumours have identified potentially important loci for genetic abnormality (Sandberg, 1992; Miyao et al, 1993; Matsuyama et al, 1994; Sauter et al, 1994; Kallioniemi et al, 1995). Losses on many chromosomes, including 17, 11, 9, 8, 5 and 3 have been associated with high stage and grade, and links have been implied to poor disease outcome (Sandberg, 1992; Miyao et al, 1993; Matsuyama et al, 1994; Sauter et al, 1994; Kallioniemi et al, 1995).

Loss of chromosome 9 loci is the most frequently observed and studied genetic event in TCC. To date, three discrete loci for genetic loss have been identified on this one chromosome. After observation that ABO blood group antigens were frequently lost in bladder cancer it was shown that loss of the distal long arm of chromosome 9 (9q34) was also frequent (22–60%). A distinct region of loss has been identified at 9q22 (Orlow et al, 1994; Habuchi et al, 1995). Recently, interest has centred on loss on chromosome 9p21 in TCC. The identification of a tumour-suppresser gene (MTS1, p16) at this locus and the high frequency of its loss in TCC (Linnenbach et al, 1993; Devlin et al, 1994; Keen and Knowles, 1994) confirm the importance of 9p21 as the site of an early bladder cancer event. Between 28% and 60% of tumours have been shown to lack copies of all chromosome 9 loci (Keen and Knowles, 1994; Orlow et al, 1994; Habuchi et al, 1995; Sauter et al, 1995) tested.

In most studies comparisons of tumour genetic events have been without reference to the patient's clinical outcome. A major diagnostic challenge in TCC is early identification of patients at risk of recurrence or progression. This study suggests that early events in tumorigenesis can provide clues to patient outcome.

*Received 4 November 1996*
*Revised 18 November 1997*
*Accepted 19 November 1997*

*Correspondence to:* JMS Bartlett, University Department of Surgery, Level II, Queen Elizabeth Building, Glasgow Royal Infirmary, Glasgow G31 2ER, UK

**Table 1** Patient demographic information

| | NR (*n* = 20) | RNP (*n* = 21) | RP (*n* = 12) |
|---|---|---|---|
| Age at diagnosis (mean ± s.d.) | 67.0 ± 8.3 | 68.5 ± 12.1 | 65.1 ± 13.2 |
| Sex M/F | 16:4 | 15:6 | 8:4 |
| Follow-up years (mean ± s.d.) | 5.1 ± 3.4 | 5.2 ± 4.2 | 4.3 ± 3.8 |
| Number of tumours per patient (mean ± s.d.) | 1 | 6.4 ± 4.2 | 6.8 ± 2.6 |
| Number of cystoscopies (mean ± s.d.) | 8.0 ± 4.3 (range 1–18) | 10.0 ± 6.3 (range 2–22) | 9.5 ± 7.7 (range 3–30) |
| Stage | | | |
| pTa | 13 (65%) | 16 (76%) | 6 (50%) |
| pT1 | 6 (30%) | 5 (24%) | 6 (50%) |
| pT2 | 1 (5%) | – | – |
| pT2+ | – | – | – |
| Grade | | | |
| 1 | 8 (40%) | 12 (58%) | 4 (33%) |
| 2 | 10 (50%) | 8 (37%) | 3 (25%) |
| 3 | 2 (10%) | 1 (5%) | 5 (42%) |
| Outcome (number dead from bladder cancer) | 0 | 0 | 5 |

NR, non-recurrent; RNP, recurrent non-progressive; RP, recurrent progressive. Age, mean age (± standard deviation) of patients in each group at first diagnosis. Follow-up, mean follow-up (years ± s.d.). Tumours, mean number of tumours per patient. TCC deaths, number of patients/group who died from TCC. pTa/pT1, distribution of stage, grade (G1–G3) for primary tumour events from each patient.

### MATERIALS AND METHODS

Three patient groups were studied. Patients with a solitary episode of superficial (pTa–pT1) TCC who showed no subsequent disease over 3 or more years of follow-up (non-recurrent group, NR). Patients with superficial TCC and subsequent, often repeated, recurrence of superficial disease but without progression to detrusor muscle invasion or metastasis (recurrent non-progressive group, RNP). Finally, patients presenting with superficial papillary TCC who subsequently progressed to invasion of the bladder wall or to metastasis (pT2–4; recurrent progressive group, RP).

Twenty NR, 21 RNP and 12 RP patients were identified for this study. All patients had their primary superifical tumours analysed. In 12 RNP and eight RP patients subsequent tumour events were also analysed.

Patients were selected for availability of TCC tissue from all primary and recurrent carcinomas; patients with diathermy without biopsy of any events before primary, recurrent TCC or the first invasive event (pT2–4), or presenting with primary invasive TCC (pT2–4) or with inadequate referral, or when material was inadequate for sampling were excluded. No significant differences in patient age ($P$ = 0.234), male–female ratio ($P$ = 0.41), mean follow-up ($P = 0.279$), number of cystoscopies ($P = 0.77$), ($P = 0.31$) or grade ($P = 0.35$) at presentation were observed between different patient groups (Table 1).

### Fluorescence in situ hybridization (FISH)

Serial 6-µm skin sections and subsequently normal bladder sections were used as positive controls within each experimental run. FISH was performed as described previously (Murphy et al, 1995). Essentially, 6 µm-sections on silane-treated slides (Sigma, UK) were dewaxed and rehydrated immediately before use. Pepsin digestion was performed at 37°C for 15–60 min. Sections were washed and post-fixed in Streck tissue fixative (Alphalabs, Cambridge, UK), dehydrated and air dried. Chromosome 9 classical satellite probe (9q12, Appligene Oncor UK) or a combination of chromosome 7 and 17 alpha satellite probes (Appligene Oncor UK) diluted in hybridization mix (50% formamide, 2 × sodium saline citrate (SSC), 0.5% salmon sperm DNA, 10% dextran sulphate, all from Sigma) were applied and sections denatured at 72°C for 5 min and incubated overnight at 37°C. After post-hybridization washes, signal was detected using sheep anti-digoxigenin (Boehringer, UK), FITC anti-sheep (Stratech, UK) for chromosome 9, and FITC avidin/biotinylated goat anti-avidin (both Vector) with rabbit anti-digoxigenin/CY3 labelled donkey anti-rabbit (both from Jackson) for chromosomes 7 and 17. Slides were mounted in Vectashield (Vector, UK) mountant containing DAPI (30 µg $ml^{-1}$, Sigma, UK) and sealed. Signals were visualized using a DMLB Microscope 100-W mercury lamp (Leica, UK). Image analysis of nuclear size confirmed that variations between tumours and skin controls were non-significant both statistically and in terms of impact on nuclear truncation (see Phalplatz et al, 1995).

### Scoring procedure

All control and carcinoma samples were scored 'blind' and independently by two observers, in each area/section epithelial cell nuclei were identified and the number of observed signals, representing hybridization of the DNA probe (0, 1, 2, 3, etc.) per nucleus, recorded using a multichannel counter for 200 nuclei per section (Figure 1). Analysis of results demonstrated that both the monosomic cell population (MCP) and the mean chromosome copy number/nucleus (MCCN) ratio were equivalent measures of monosomy 9 (data not shown). For each area of tissue, values were calculated for both observers, and the interobserver mean calculated as the final value for the section in question.

Multiple areas of carcinoma (range 1–6, median 3) were scored for each carcinoma and analysed as described above to ensure scoring results were consistent in all tumour samples. Tumour areas were scored by each observer repeatedly (two to four times)

© Cancer Research Campaign 1998

**Figure 1** Illustrative example of scoring histogram from four tumours, Scoring data from two observers for four tumour areas. Number of cells scored (vertical axis) with 0, 1, 2 or 3 chromosome signals (horizontal axis). ▨, ▧ represent two monosomic tumours for chromosome 9; ▥, ▤ two disomic tumours for chromosome 9. Monosomic tumours show predominantly cells with single copies, whereas disomic tumours show predominantly two copies of chromosome 9

**Figure 2** (**A**) Photomicrograph of tumour biopsy showing areas of tumour selected for analysis. (**B**) Fluorescence photomicrograph of normal skin epithelium. Note disomic signal pattern throughout. (**C**) Fluorescence photomicrograph of TCC bladder epithelium with no loss of chromosome 9. Note disomic signal pattern throughout. (**D**) Fluorescence photomicrograph of TCC bladder epithelium with loss of chromosome 9. Note loss of signals throughout

© Cancer Research Campaign 1998

on separate occasions to evaluate intraobserver variation. Interobserver variation between areas was calculated using mean values from observer A and observer B for each area of tumour scored. Overall interobserver variation was calculated as the mean variation for all areas evaluated.

### Statistics

Differences between multiple groups were analysed using ANOVA (in the case of parameters such as age, length of follow-up, etc.) or chi-squared tests (for stage, grade, sex ratios, etc.) as appropriate. Survival was analysed using Kaplan–Meier analysis with the log-rank test.

### RESULTS

### Patients

There were no significant differences in any measured parameters between patients at recruitment into the different study groups. By definition, the progress of disease in each of the patient groups showed marked differences. Strikingly, numbers of cystoscopies per patient were not significantly different between patients with NR and recurrent disease (Table 1). The probability of dying from any cause was significantly greater in recurrent patients ($P$ = 0.0402). The probability of dying from bladder cancer ($P$ = 0.0032) was significantly higher for RP patients as all TCC-related deaths were in progressed patients.

### Fluorescence in situ hybridization

Nuclear diameters for control sections were 7.4 μm ± 12.5%, the range for tumour median diameters was 6.68–9.04 μm. Calculations based on the method of Phalplatz et al (1995) suggest that the maximum error, relating to a hypothetical change in nuclear size between control samples and tumours would be 10.12%, well within the detection criteria of this technique. However, this result would suggest that for detection of polyploidies above $n = 6$ a potential for type II sampling error between the control and the tumour samples exists. Samples analysed showed clear hybridization at two loci in the majority of nuclei from skin sections (Figure 2B). Areas of tumour from patients were either uniformly similar to skin in appearance (Figure 2C) or showed a high proportion of signal loss (Figure 2D).

The results of analysis of replicate scoring of either 24 sections (observer A) or 35 sections (observer B) demonstrated that the mean chromosomal copy number showed greater consistency of reporting with mean intraobserver variation of 11.91% (8.01–15.81%) than the percentage monosomic cell population. For both chromosomes normal ranges were defined as the mean chromosomal copy number ± 3 × s.d. for values obtained for control tissues. For chromosome 9 the normal range was 1.51–2.10 and for chromosomes 7 and 17 the normal range was 1.37–1.85; this reflects the lower hybridization efficiency of these probes. MCCN values obtained for tumour samples incorporate a 4.00% or 6.00% (chromosomes 9 and 7 or 17 respectively) observer error that was taken into account when defining abnormalities.

In all, 135 carcinoma areas were scored by both observers, mean interobserver variation for MCP was 15.02% (median 11.19%, maximum 59.42%), whereas the mean interobserver variation for chromosomal copy number was 4.00% (median 3.57%, maximum 15.58%). In the majority of cases (96.0%) tissue chromosome 9 copy numbers were consistent in all areas of tumour evaluated; in only three tumours was heterogeneity of copy number at this locus identified. In one RNP patient the first recurrence demonstrated one tumour area with a mean chromosomal copy number of 0.9 for chromosome 9, the final recurrence from this patient showed consistent monosomy throughout. Secondly, in one RP patient, the primary tumour was monosomic in two out of four areas, with a mean chromosomal copy number of 1.02. Both these patients were clearly classified as monosomic. Finally, in one NR patient a single area out of four scored showed a mean chromosomal copy number of 1.37, with other areas showing a mean copy number of 1.75. This result falls outwith the range observed for both monosomic and disomic patients, such that this area cannot be truly classified as either monosomic or disomic and has been excluded from analysis.

### Chromosome 9 monosomic tumours/patients

Of 53 primary tumours analysed for chromosome 9 copy number, 13 were monosomic and 40 disomic for chromosome 9 copy number. Overall, monosomy for this locus could be clearly demonstrated in 1 out of 20 NR (5%) and 12 out of 32 (36%) of *primary* recurrent (either RNP or RP) tumours ($P$ = 0.0085; Figure 3). Of all patients, 1 out of 20 NR and 13 out of 32 (41%) of recurrent patients demonstrated monosomy during the course of follow-up ($P$ = 0.0048). No difference in the rate of monosomy of tumours was apparent between RNP or RP patients.

Monosomy for the chromosome 9 classical satellite detected in the primary tumour from a patient was clearly associated with

**Figure 3** Mean chromosomal copy number (vertical axis) in primary tumours from patients with non-recurrent (NR), recurrent non-progressive (RNP) and recurrent progressive (RP) disease. One out of 20 non-recurrent tumours and 12 out of 33 recurrent (RNP + RP) patients showed monosomy for chromosome 9 in their primary tumours

© Cancer Research Campaign 1998

disease recurrence ($P = 0.0085$) and also with monosomy of all subsequent tumours. No significant difference in the rates of monosomy of primary tumours was observed between RNP and RP patients (Figure 2).

Of 104 TCC analysed, 28 (28%) were monosomic throughout. Of superficial TCCs (pTa–pT1) 24.6% were monosomic vs 44.4% of advanced (pT2 + $n$ = 9) TCCs. When segregated by grade, 28.6% of G1, 41.7% of G2 and 26.7% of G3 tumours were monosomic, in that order. No relationship between monosomy and tumour stage or grade was identified. Of the 28 monosomic tumours, two represented the last known event from two separate RP patients (2 out of 12, 16.7%), whereas the remaining 26 tumours represented all tumour events analysed, including the primary TCC, from a further 12 patients (seven RNP patients (33%) and five RP patients (42%) (Figure 4)) . A single NR patient showed monosomy.

### Chromosome 7 and 17

A total of 53 primary tumours were scored for chromosome 7 and 17 copy number. All 20 NR patients showed normal copy numbers for chromosomes 7 and 17 (chromosome 7 mean 1.73, range 1.61–1.87, 17 mean 1.71, range 1.51–1.91). Of 33 primary tumours from recurrent/RP patients 25 exhibited normal copy numbers for chromosome 17. The remaining eight tumours (24%) showed significant elevations in MSNR (range 2.01–2.66); of these, six showed elevations in MSNR for chromosome 7 (range 1.98–2.71). No significant relationship between abnormalities of chromosome 7 or 17 and stage (either pTa or pT1) or grade was observed. No relationship between monosomy chromosome 9 and polysomy 17 was observed. A modestly significant link between polysomy 7 and 17 and recurrence could be established ($P = 0.04$, chromosome 17, $P = 0.015$ chromosome 7).

### DISCUSSION

Analysis of chromosome 9 copy number using the pericentromeric classical satellite at 9q12 has been widely used in studies of TCC of the bladder (Matsuyama et al, 1994; Orlow et al, 1994; Sauter et al, 1995). By identifying patients with known disease outcome, this study demonstrates that loss of this locus, and presumably of the whole chromosome, implies elevated risk of recurrent TCC. The consistency of this loss and its early occurrence may provide a predictive marker of disease outcome in these patients.

Most studies of molecular events in TCC have evaluated relationships between tumour stage and grade. The current investigation has investigated genetic events within TCC and disease outcome. To achieve this aim, patients were selected and grouped by outcome as defined above. Three patient groups were evaluated: those with single superficial (pTa/pT1) TCC (NR patients), those whose TCC recurred without progression (RNP patients) and those who progressed to invasive TCC at recurrence (RP patients). This patient-based evaluation focuses on molecular mechanisms underlying these clinically distinct disease processes that can be exploited for diagnosis or therapy.

Patients in the three groups were similar in age, sex ratio and follow-up, including numbers of cystoscopies. Analysis of the primary TCC from these patients showed a relationship between disease outcome and high grade (G1 vs G2 + 3) but not stage (Table 1).

**Figure 4** Chromosomal copy number for chromosome 9 in recurrent tumours. Mean chromosomal copy number (vertical axis) in recurrent tumours from representative patients from both RNP and RP groups. Tumours analysed were primary, first identified superficial TCC for all patients; 1st recurrence, first recurrence in all patients (also superficial); preinvasion, last identified tumour for RNP and last non-invasive (pTa/pT1) tumour for RP patients; post-invasion, for RP patients only, first diagnosed invasive tumour (pT2+). △, ▲, two RNP patients (BLA32 and BLA33) with monosomy 9 in all tumours analysed; ○, ●, two RNP patients (BLA92 and BLA145) with disomy 9 in all tumours analysed; ■, □, two RP patients with Monosomy 9 in all tumours analysed (BLA45 and BLA135); ▽, ▼, two RP with disomy 9 in all tumours analysed (BLA3 and BLA12), ◆, ◇, 2 RNP patients with disomy 9 in their primary tumours who subsequently demonstrated monosomy 9 (BLA40 and BLA78)

The FISH method used to quantify chromosomal copy number in situ has been critically evaluated. Scoring was performed independently by two observers and variation between observers shown to be consistently low for the MCCN (8–15%). Essentially, the parameters investigated are interchangeable (Figure 1), although MCCN is consistently less variable than MCP.

An interesting finding was the consistency of genetic alterations, not only within tumour samples taken at a single time point but also across TCCs from the same patients many years apart. This contrasts with high rates of tumour heterogeneity reported in other studies for different loci. Although some loci may show spatial and temporal heterogeneity in tumour cells, this is a significant finding in TCC for this chromosome 9 locus. Other loci show heterogeneity in TCC (Schapers et al, 1993), but this is not a uniform finding (Oliver et al, 1992). It appears that markers of tumour behaviour may exist that are not complicated by problems of intratumour heterogeneity.

In addition to the observation that loss of chromosome 9 is consistent in multiple samples of single TCC tumours, it is clear that such loss occurs early and persists when multiple TCCs from single patients are evaluated. Of the 20 patients evaluated over time at this locus, only two showed a change in chromosomal copy number during the course of their disease, in both this was a later event. This demonstrates that although early loss of chromosome 9 is usual in TCC, later changes cannot be ruled out.

Arguably the finding that genetic changes in primary TCC may predict those in subsequent tumours is of greatest diagnostic interest. We have previously identified c-erbB-2 gene amplification in TCC as a marker of invasion and poor survival (Underwood et al, 1995), but c-erbB-2 amplification failed to predict invasion early enough to allow for a modification in patient management. In

© Cancer Research Campaign 1998

this context, the consistency of chromosome 9 losses between primary tumours and recurrences more than 5 years later suggests that diagnostic markers of value may exist. We have investigated the relationship between monosomy 9 and chromosomes 7 and 17, solely in primary tumours, to determine whether the lesion on chromosome 9 is specific. No alterations in copy number for chromosomes 7 and 17 tested was observed in NR patients. Lower than expected frequencies of polysomy 7 and 17 were observed in the primary tumours from recurrent patients (8 out of 33; 24%). Abnormalities in chromosomes 7 and 17 were confined in this study to patients destined to recur, and there was a modest link between polysomy 7 or 17 and recurrences ($P$ = 0.04 and 0.02 respectively). Other studies have identified higher rates of polysomy for 7 and 17 in bladder tumours; our finding may suggest, as is highlighted elsewhere, that aberrations in these chromosomes occur later in the disease process (Sandberg, 1992; Schapers et al, 1993; Matsuyama et al, 1994; Sauter et al, 1995; Resnikoff et al, 1996). We have selectively studied primary superficial tumours in this cohort rather than recurrent and progressive (pT2 +) tumours in previous reports. There is therefore a marked difference in our tumour cohort from those in previous studies, and this difference may explain differences in rates of aneusomy 7 and 17 observed.

The relationship between disease recurrence and chromosome 9 may suggest the existence of a 'recurrence'-related gene on this chromosome, the identification of which may in future provide a therapeutic target. The specific loci that have been previously identified as sites of loss in TCC that are candidates for this 'recurrence' gene remain to be evaluated. It may be that such a gene is not in itself related to recurrence but rather in a manner analagous to that for p53, may allow or be associated with further genetic changes that result in recurrence.

This study identifies chromosome 9 loss as a possible predictive marker for disease recurrence in TCC patients. Analysis of patient outcome in this multistage disease shows a relationship between loss of chromosome 9, measured by FISH of the 9q12 classical satellite, in primary tumours of patients and disease recurrence. Wider prospective or retrospective analysis is required to confirm the usefulness of this marker for early identification of patients with superficial TCC at risk of recurrence and therefore progression. This study validates the search for molecular markers of disease outcome in TCC. Subchromosomal studies of chromosome 9 may localize a potential 'recurrence' gene and increase the range of patients for whom this test may be of value.

### REFERENCES

Anonymous (1987) Office of Population Censuses and Studies DH1. 1969–1987, HMSO: London

Anonymous (1984) Office of Population Censuses and Studies MB1. 1971–1984, HMSO: London

Devlin J, Keen AJ and Knowles MA (1994) Homozygous deletion mapping at 9p21 in bladder carcinoma defines a critical region within 2cM of IFNA. *Oncogene* **9**: 2757–2760

Habuchi T, Devlin J, Elder PA and Knowles MA (1995) Detailed deletion mapping of chromosome 9q in bladder cancer: evidence for two tumour suppressor loci. *Oncogene* **11**: 1671–1674

Kallioniemi A, Kallioniemi OP, Citro G, Sauter G, De Vries S, Kerschmann R, Caroll P and Waldman F (1995) Identification of gains and losses of DNA sequences in primary bladder cancer by comparative genomic hybridization. *Genes Chromosom Cancer* **12**: 213–219

Keen AJ and Knowles MA (1994) Definition of two regions of deletion on chromosome 9 in carcinoma of the bladder. *Oncogene* **9**: 2083–2088

Linnenbach AJ, Pressler LB, Seng BA, Kimmel BA, Tomaszewski JE and Malkowicz SB (1993) Characterization of chromosome 9 deletions in transitional cell carcinoma by microsatellite assay. *Human Mol Genet* **2**: 1407–1411

Matsuyama H, Bergerheim USR, Nilsson I, et al (1994) Nonrandom numerical aberrations of chromosomes 7, 9, and 10 in DNA-diploid bladder cancer. *Cancer Genet Cytogenet* **77**: 118–124

McCredie M (1994) Bladder and kidney cancers. *Cancer Surveys* **19–20**: 343–368

Miyao N, Tsai YC, Lerner SP, Olumi AF, Spruck CH, Gonzalez Zulueta M, Nichols PW, Skinner DG and Jones PA (1993) Role of chromosome 9 in human bladder cancer. *Cancer Res* **53**: 4066–4070

Murphy DS, McHardy P, Coutts J, Mallon EA, George WD, Kaye SB, Brown R and Keith WN (1995) Interphase cytogenetic analysis of erbB2 and topoIIalpha co-amplification in invasive breast cancer and polysomy of chromosome 17 in ductal carcinoma in situ. *Int J Cancer* **64**: 18–26

Oliver RTD, Feldman DN, Sidransky D, Vogelstein B, Frost P, Von Eschenbach AC, Neal DE and Harris AL (1992) Monoclonality of multiple bladder cancers (3). *N Engl Med* **327**: 433–434

Orlow I, Lianes P, Lacombe L, Dalbagni G, Reuter VE and Cordoncardo C (1994) Chromosome 9 allelic losses and microsatellite alterations in human bladder tumors. *Cancer Res* **54**: 2848–2851

Ozen H (1994) Transitional cell carcinoma of the bladder. *Curr Opin Oncol* **6**: 313–317

Phalplatz MMM, Dewilde PCM, Poddighe P, Dekken HV, Vooijs GP and Hanselaar AGJM (1995) A model for evaluation of in situ hybridization spot-count distributions in tissue sections. *Cytometry* **20**: 193–202

Reznikoff CA, Belair CD, Yeager TR, Savelieva E, Blelloch RH, Puthenveettil JA and Cuthill SA (1996) A molecular genetic model of human bladder cancer pathogenesis. *Semin Oncol* **23**: 571–584

Sandberg AA (1992) Chromosome changes in early bladder neoplasms. *J Cell Biochem* **50**: 76–79

Sauter G, Deng G, Moch H, Kerschmann R, Matsumura K, De Vries S, George T, Fuentes J, Carroll P and Mihatsch MJ (1994) Physical deletion of the p53 gene in bladder cancer. Detection by fluorescence in situ hybridization. *Am J Pathol* **144**: 756–766

Sauter G, Moch H, Carroll P, Kerschmann R, Mihatsch MJ and Waldman FM (1995) Chromosome-9 loss detected by flourescence in situ hybridization in bladder cancer. *Int J Cancer* **64**: 99–103

Schapers R, Smeets W, Hopman A, Pauwels R, Geraedts J and Remaekers F (1993) Heterogeneity in bladder cancer as detected by conventional chromosome analysis and interphase cytogenetics. *Cancer Genet Cytogenet* **70**: 56–61

UICC. (1978) TNM classification of malignant tumours. 1978; Wiley-Liss: New York

Underwood M, Bartlett J, Reeves J, Gardiner DS, Scott R and Cooke T (1995) C-erbB-2 gene amplification: a molecular marker in recurrent bladder tumors? *Cancer Res* **55**: 2422–2430

*© Cancer Research Campaign 1998*


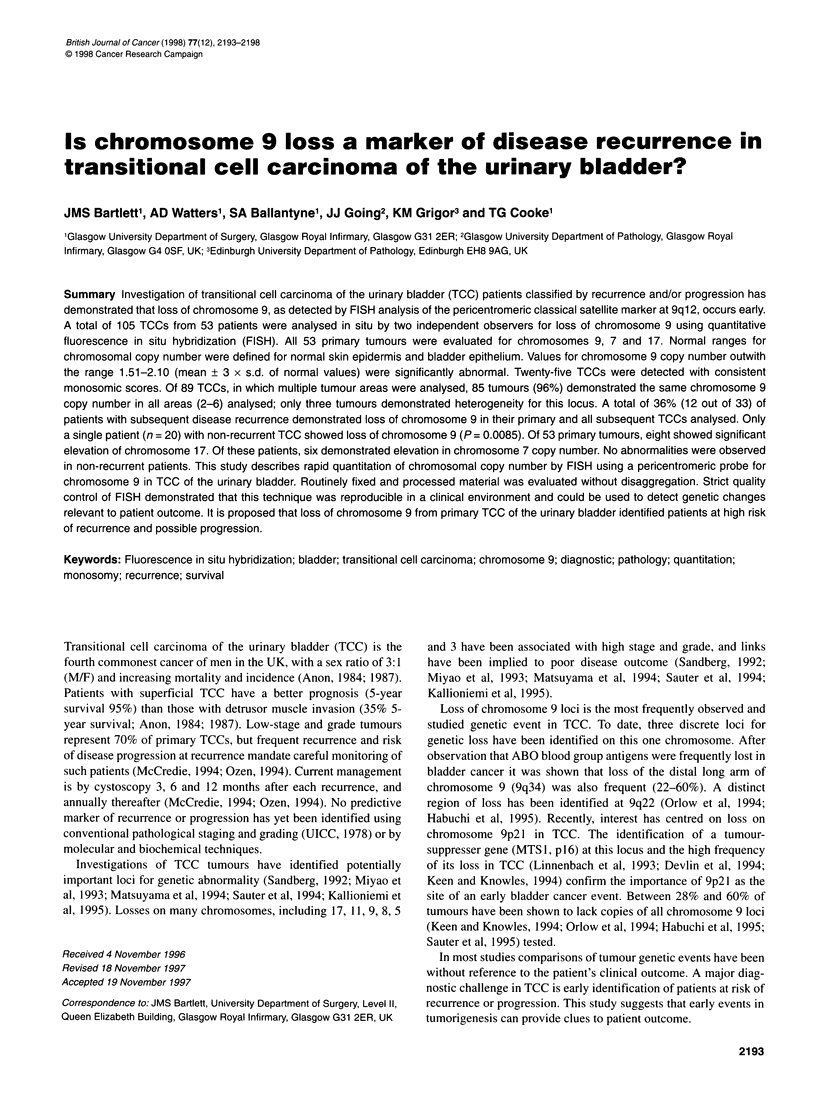

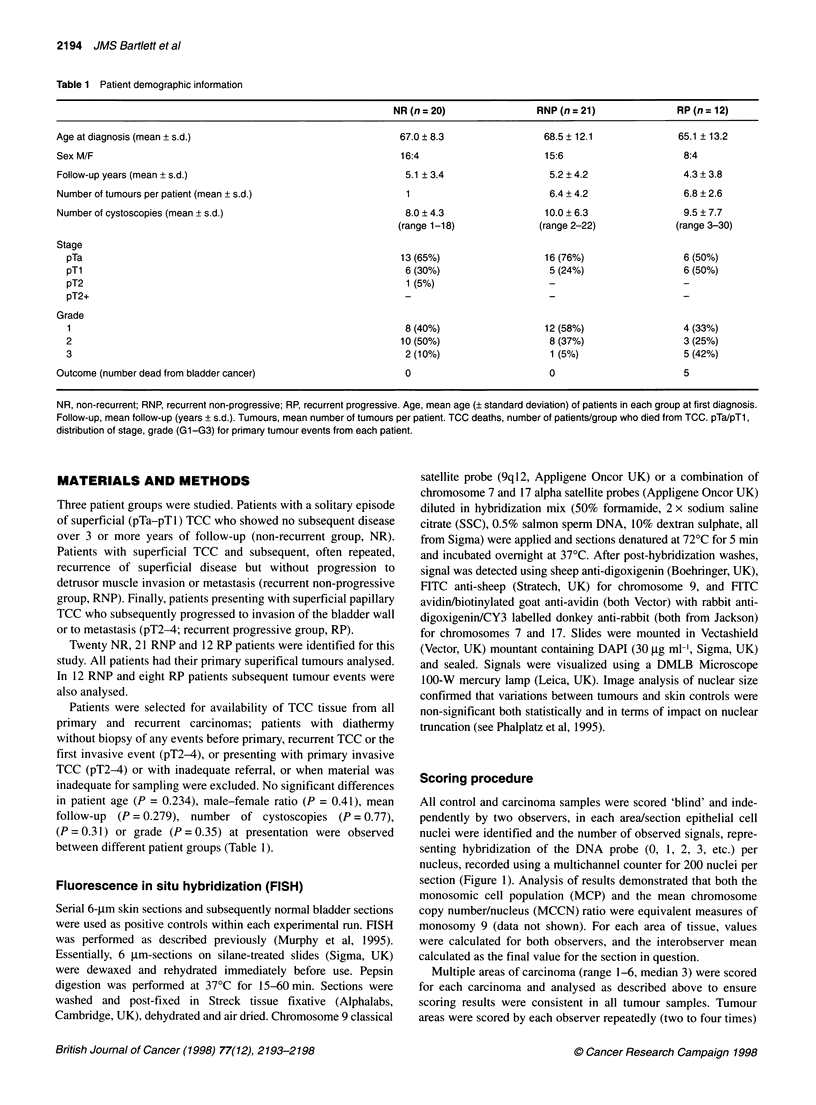

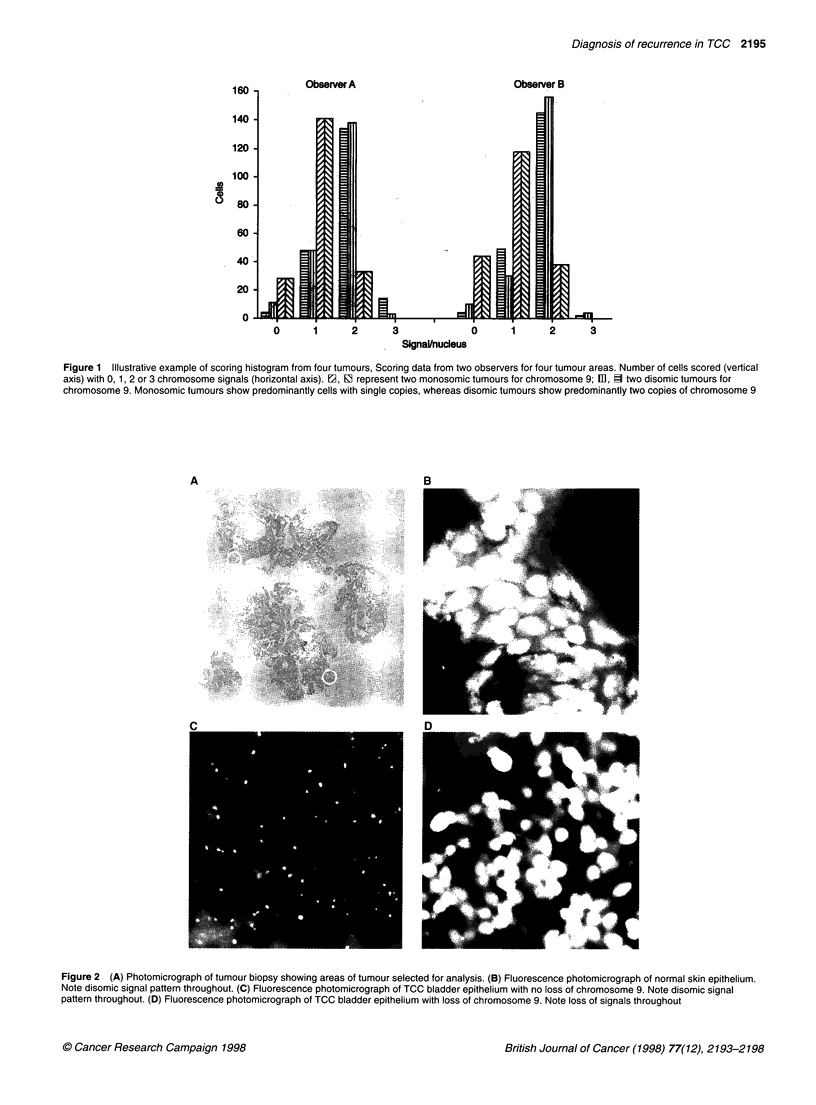

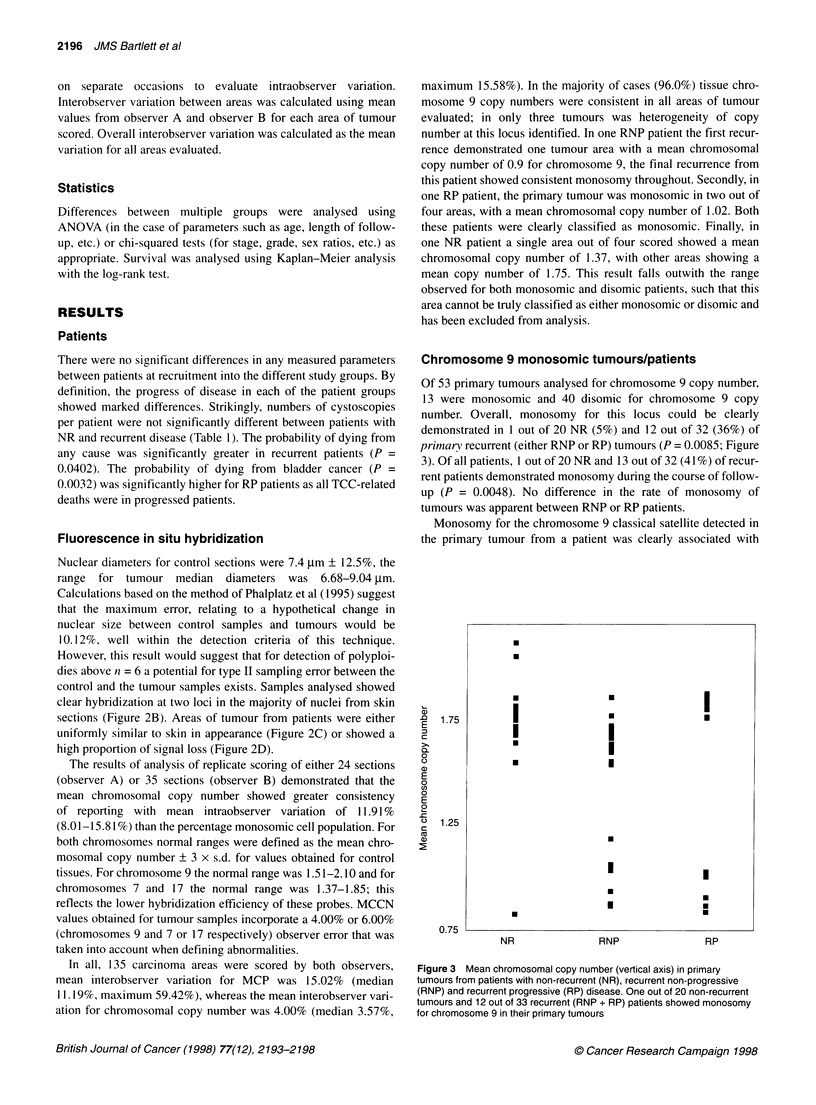

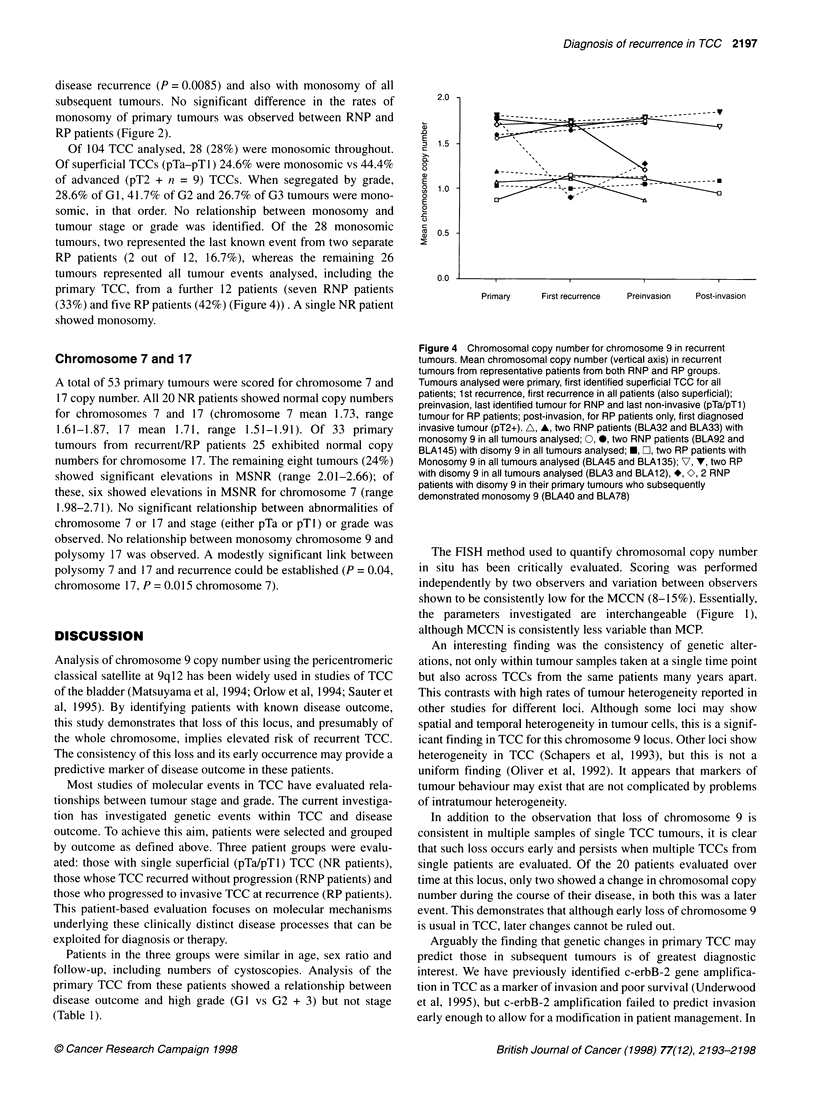

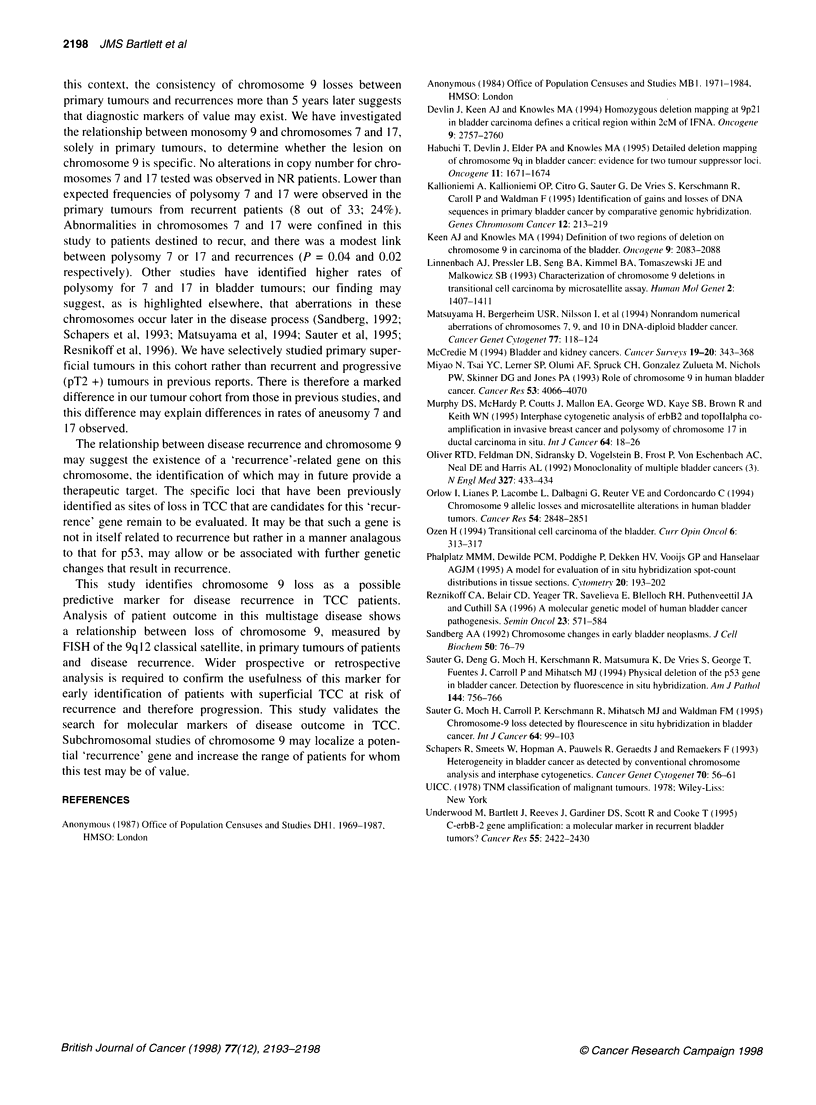


## References

[OCR_00627] Devlin J., Keen A. J., Knowles M. A. (1994). Homozygous deletion mapping at 9p21 in bladder carcinoma defines a critical region within 2cM of IFNA.. Oncogene.

[OCR_00634] Habuchi T., Devlin J., Elder P. A., Knowles M. A. (1995). Detailed deletion mapping of chromosome 9q in bladder cancer: evidence for two tumour suppressor loci.. Oncogene.

[OCR_00637] Kallioniemi A., Kallioniemi O. P., Citro G., Sauter G., DeVries S., Kerschmann R., Caroll P., Waldman F. (1995). Identification of gains and losses of DNA sequences in primary bladder cancer by comparative genomic hybridization.. Genes Chromosomes Cancer.

[OCR_00644] Keen A. J., Knowles M. A. (1994). Definition of two regions of deletion on chromosome 9 in carcinoma of the bladder.. Oncogene.

[OCR_00649] Linnenbach A. J., Pressler L. B., Seng B. A., Kimmel B. S., Tomaszewski J. E., Malkowicz S. B. (1993). Characterization of chromosome 9 deletions in transitional cell carcinoma by microsatellite assay.. Hum Mol Genet.

[OCR_00659] McCredie M. (1994). Bladder and kidney cancers.. Cancer Surv.

[OCR_00661] Miyao N., Tsai Y. C., Lerner S. P., Olumi A. F., Spruck C. H., Gonzalez-Zulueta M., Nichols P. W., Skinner D. G., Jones P. A. (1993). Role of chromosome 9 in human bladder cancer.. Cancer Res.

[OCR_00666] Murphy D. S., McHardy P., Coutts J., Mallon E. A., George W. D., Kaye S. B., Brown R., Keith W. N. (1995). Interphase cytogenetic analysis of erbB2 and topoII alpha co-amplification in invasive breast cancer and polysomy of chromosome 17 in ductal carcinoma in situ.. Int J Cancer.

[OCR_00686] Pahlplatz M. M., de Wilde P. C., Poddighe P., van Dekken H., Vooijs G. P., Hanselaar A. G. (1995). A model for evaluation of in situ hybridization spot-count distributions in tissue sections.. Cytometry.

[OCR_00691] Reznikoff C. A., Belair C. D., Yeager T. R., Savelieva E., Blelloch R. H., Puthenveettil J. A., Cuthill S. (1996). A molecular genetic model of human bladder cancer pathogenesis.. Semin Oncol.

[OCR_00696] Sandberg A. A. (1992). Chromosome changes in early bladder neoplasms.. J Cell Biochem Suppl.

[OCR_00702] Sauter G., Deng G., Moch H., Kerschmann R., Matsumura K., De Vries S., George T., Fuentes J., Carroll P., Mihatsch M. J. (1994). Physical deletion of the p53 gene in bladder cancer. Detection by fluorescence in situ hybridization.. Am J Pathol.

[OCR_00706] Sauter G., Moch H., Carroll P., Kerschmann R., Mihatsch M. J., Waldman F. M. (1995). Chromosome-9 loss detected by fluorescence in situ hybridization in bladder cancer.. Int J Cancer.

[OCR_00711] Schapers R., Smeets W., Hopman A., Pauwels R., Geraedts J., Ramaekers F. (1993). Heterogeneity in bladder cancer as detected by conventional chromosome analysis and interphase cytogenetics.. Cancer Genet Cytogenet.

[OCR_00719] Underwood M., Bartlett J., Reeves J., Gardiner D. S., Scott R., Cooke T. (1995). C-erbB-2 gene amplification: a molecular marker in recurrent bladder tumors?. Cancer Res.

